# Folding machineries displayed on a cation-exchanger for the concerted refolding of cysteine- or proline-rich proteins

**DOI:** 10.1186/1472-6750-9-27

**Published:** 2009-03-26

**Authors:** Dae-Hee Lee, Sung-Gun Kim, Dae-Hyuk Kweon, Jin-Ho Seo

**Affiliations:** 1 Department of Agricultural Biotechnology, Seoul National University, Seoul 151-921, Korea; 2 School of Biotechnology and Bioengineering, Sungkyunkwan University, Suwon 440-746, Korea; 3 Current address : Department of Bioengineering, University of California San Diego, La Jolla, CA 92093, USA; 4 Current address : Department of Biochemistry, Robert Wood Johnson Medical School, Piscataway, NJ 08854, USA

## Abstract

**Background:**

*Escherichia coli *has been most widely used for the production of valuable recombinant proteins. However, over-production of heterologous proteins in *E. coli *frequently leads to their misfolding and aggregation yielding inclusion bodies. Previous attempts to refold the inclusion bodies into bioactive forms usually result in poor recovery and account for the major cost in industrial production of desired proteins from recombinant *E. coli*. Here, we describe the successful use of the immobilized folding machineries for *in vitro *refolding with the examples of high yield refolding of a ribonuclease A (RNase A) and cyclohexanone monooxygenase (CHMO).

**Results:**

We have generated refolding-facilitating media immobilized with three folding machineries, mini-chaperone (a monomeric apical domain consisting of residues 191–345 of GroEL) and two foldases (DsbA and human peptidyl-prolyl *cis-trans *isomerase) by mimicking oxidative refolding chromatography. For efficient and simple purification and immobilization simultaneously, folding machineries were fused with the positively-charged consecutive 10-arginine tag at their C-terminal. The immobilized folding machineries were fully functional when assayed in a batch mode. When the refolding-facilitating matrices were applied to the refolding of denatured and reduced RNase A and CHMO, both of which contain many cysteine and proline residues, RNase A and CHMO were recovered in 73% and 53% yield of soluble protein with full enzyme activity, respectively.

**Conclusion:**

The refolding-facilitating media presented here could be a cost-efficient platform and should be applicable to refold a wide range of *E. coli *inclusion bodies in high yield with biological function.

## Background

*Escherichia coli *retains its dominant position as the first choice of host for the high-throughput production of proteins of therapeutic or commercial interest because of rapid biomass accumulation, well-established genetic manipulation methods and simple process scale-up [1,2]. However, a major disadvantage derived from the intrinsic properties of *E. coli *is the frequent formation of an inclusion body, a dense aggregate of misfolded polypeptides [3]. Refolding the inclusion bodies into the native conformation might be straightforward if an efficient refolding scheme is established. There are a variety of conventional methodologies for refolding recombinant proteins from inclusion bodies, which contains simple dilution, dialysis and chromatographic refolding methods. Dilution is the simplest and most widely used technique and involves refolding initiation by reducing the denaturant concentration. However, the target product in dilution refolding can be obtained only very low yield because of protein aggregation at high protein concentration. Column-based refolding is the most likely to renaturate the protein aggregates at high concentration and to reduce the cost for reagents and buffers in large-scale protein refolding [4]. Although these conventional methods are commonly used for protein refolding and quite progress has been made, disulfide bond formation and peptidyl-prolyl *cis-trans *isomerization have often frustrated the successful refolding of insoluble recombinant proteins containing cysteine and proline in multiple numbers [5,6]. Generation of correct disulfide bonds and peptidyl-prolyl *cis-trans *configuration are required processes for formation and stabilization of the native protein conformation. Furthermore, the kinetics and thermodynamics of disulfide bond formation and peptidyl-prolyl *cis-trans *isomerization can dominate the rate and pathway of protein folding and in turn determine the refolding efficiency [7,8].

At least two classes of cellular factors are currently known to assist *in vivo *or *in vitro *protein folding. The first class includes a number of proteins collectively known as "molecular chaperones" such as GroEL-GroES and DnaK/DnaJ/GrpE in *E. coli*. Molecular chaperones are believed to prevent undesirable folding pathways such as premature folding, aggregation and misfolding and thereby facilitate formation of biologically active protein structures [9]. The second class contains foldases that catalyze protein folding by stimulating rate-limiting reactions such as the formation and interchange of disulfide bonds and isomerization of peptidyl-prolyl bonds [9]. The respective enzymes are protein disulfide bond isomerase and peptidyl-prolyl *cis-trans *isomerase (PPIase). Recently, the application of these proteins in helping *in vitro *protein refolding has been studied actively [10,11]. GroEL monomer is composed of three domains: an apical domain that is responsible for binding substrate [12], an equatorial domain that contains the ATP-binding site, and an intermediate hinge domain that connects the apical and equatorial domains. The monomeric apical domain of GroEL (mini-chaperone or mini-GroEL) was proved to trigger the *in vitro *refolding of rhodanase and cyclophilin A without GroES and ATP and had the activity of intact GroEL *in vivo *[12]. The immobilized mini-chaperone and foldases were successfully employed in column chromatography and batchwise mode to refold indole 3-glycerol phosphate synthase, cyclodextrin glycosyltransferase, antibody fragments and the scorpion toxin Cn5 in high yield with biological activity [13-16].

Here, we report a simple method that enables one-step purification and immobilization of folding machineries (mini-chaperone, DsbA and human PPIase) simultaneously in fully functional forms using the positively-charged consecutive 10-arginine tag at their C-terminal. Further, the folding-catalytic matrices immobilized with mini-chaperone and/or two foldases were recruited to refold two denatured and reduced model proteins containing multiple cysteine or proline residues for proving their refolding-assisting efficiency. This report provides an affordable refolding method using immobilized folding machineries to lead the rapid provision of the protein of interest from inclusion bodies produced in *E. coli*.

## Results

### Preparation of folding machineries

**Figure 1:**
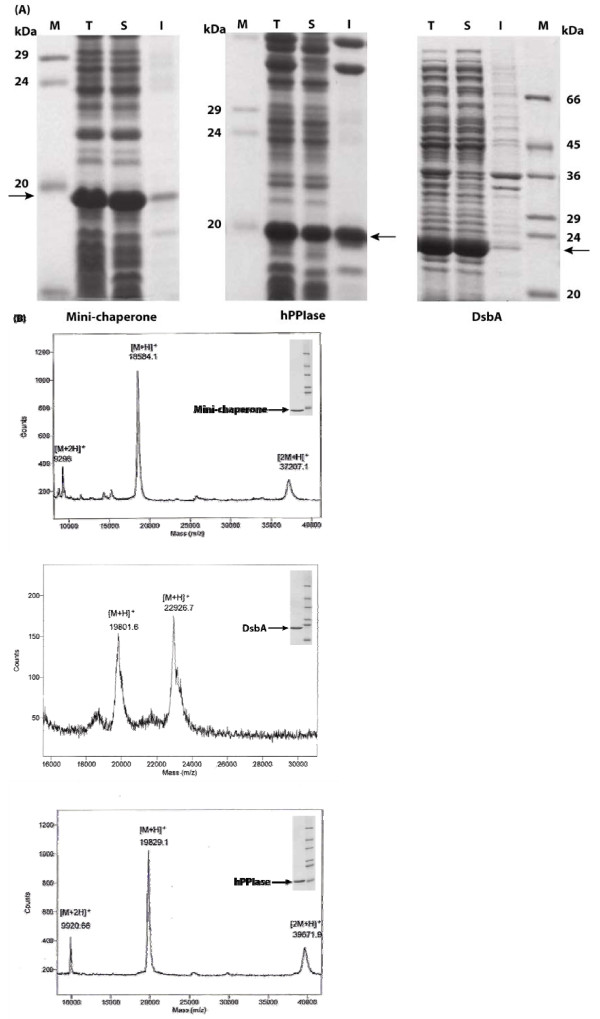
**Production of folding machineries from recombinant *E. coli***. (A) SDS-PAGE analysis of the expression of Arg_10_-tagged folding machineries in *E. coli *cultures incubated at 37°C for 6 hr and induced by 0.5 mM IPTG. I, insoluble fraction; M, molecular mass marker; S, soluble fraction; T, total cell lysates. (B) MALDI-TOF analysis of folding machineries purified by a simple ion-exchange chromatography. Mass spectra of purified folding machinery proteins: 3 (mini-chaperone and hPPIase) or 1 (DsbA) ionization states that match the molecular mass prediction of each folding machinery protein. The expressed and purified folding machinery proteins are indicated by arrows.

In order to obtain the recombinant folding machineries in *E. coli*, the PCR product coding for mini-chaperone, DsbA or human PPIase (hPPIase) was cloned into template vector harnessing the Arg_10 _tag. Mini-chaperone and DsbA expressed in *E. coli *BL21(DE3) were completely soluble and active form while approximately 50% of the total hPPIase protein was soluble and the rest of the protein was in the insoluble fraction (Fig. 1A). The recombinant *E. coli *BL21(DE3) cells transformed with the corresponding expression plasmid of folding machineries produced mini-chaperone, DsbA or hPPIase as soluble protein in final yield of about 0.41, 0.45 and 0.32 g/L of culture, respectively. After simple one-step salt-gradient ion-exchange chromatography using soluble *E. coli *cell extracts, Arg_10_-tailed folding catalysts were purified to more than 95% estimated from the sodium dodecyl sulfate polyacrylamide gel electrophoresis (SDS-PAGE) analysis (Fig. 1B, inner panel) and their predicted molecular weight were verified by matrix assisted laser desorption-ionization and time of flight (MALDI-TOF) mass spectrometry (Fig. 1B). The simply and efficiently purified folding machineries with Arg_10_-tails at their C-terminal were used for subsequent immobilization and refolding experiments.

### Construction of refolding matrices

**Figure 2:**
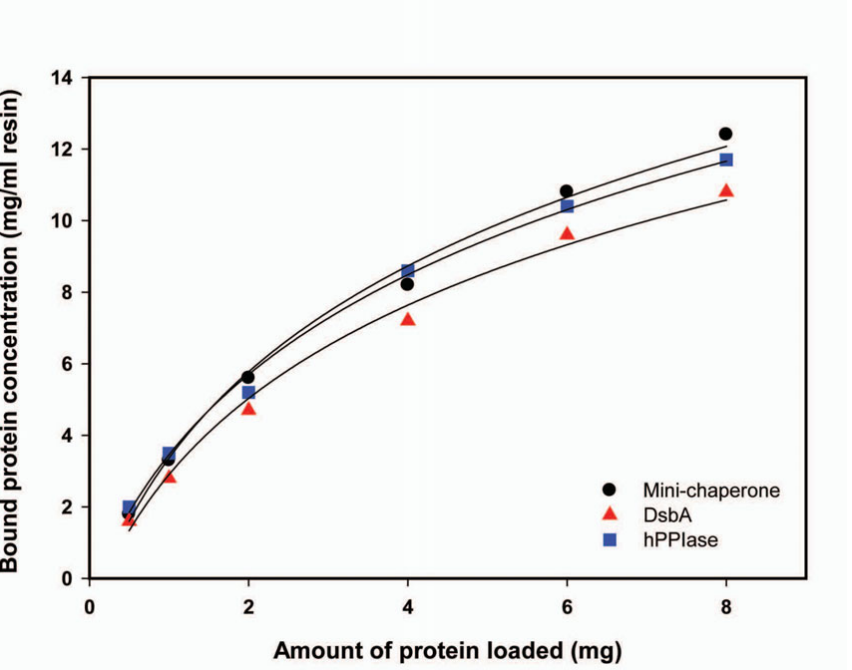
**Coupling density of folding machineries to a cation exchanger**. Various amounts of purified folding machineries were mixed with the cation exchanger, SP Sepharose Fast Flow. The folding machineries-bound SP Sepharose was mixed with 2 bead volume of 50 mM sodium phosphate buffer, pH 8.4 containing 1 M NaCl. After gentle mixing for 30 min at 25°C, the bead was removed by centrifugation and the supernatant was then used to the determination of protein concentration.

Initially, the SP-Sepharose Fast Flow resin was used to immobilize purified folding machineries for preparative purposes. Due to the stretch of 10 consecutive positively charged Arg residues of folding machineries, the purified fusion protein was immobilized noncovalently by polyionic interaction to the matrix containing polyanion as a functional group, which yielded mini-chaperone-Sepharose, DsbA-Sepharose and hPPIase-Sepharose. To measure coupling density, various amounts of purified folding machineries were mixed with the cation exchanger. The amount of immobilized folding machineries was increased as the loading amount of folding machineries into the cation exchanger was increased (Fig. 2). The maximum amount of the folding machineries coupled to the resin as follows: mini-chaperone, 12.2 mg/mL gel; DsbA, 10.8 mg/mL gel; hPPIase, 11.7 mg/mL gel. Binary (mini-chaperone/DsbA-, mini-chaperone/hPPIase- and DsbA/hPPIase-Sepharose) and ternary (mini-chaperone/DsbA/hPPIase-Sepharose) refolding matrices were prepared by thoroughly mixing in equal molar ratios of the single refolding gel (mini-chaperone-Sepharose, DsbA-Sepharose and hPPIase-Sepharose).

### Functional analyses of immobilized folding machineries

**Figure 3:**
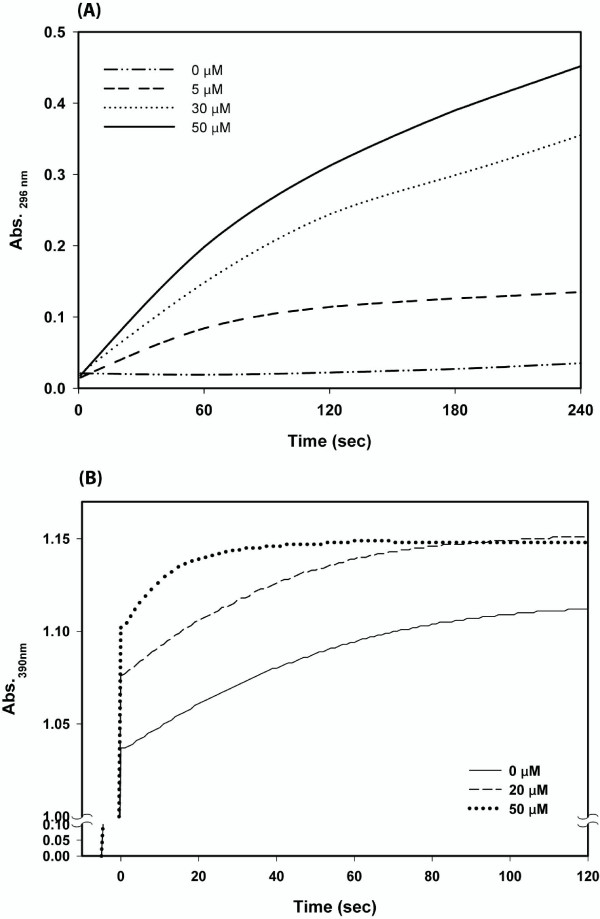
**Functional analysis of immobilized folding machineries, DsbA and hPPIase**. DsbA (A) and hPPIase (B) assay were based on their enzymatic properties to catalyze protein disulfide bond formation and *cis-trans *interconversion of the peptidyl-prolyl bond, respectively. RNase A with scrambled disulfide bonds and N-Suc-AAPF-*p*-nitroanilide were used for determination of activities of immobilized DsbA and hPPIase as substrate as described in Methods section.

Immobilized mini-chaperone was fully functional in refolding a typical substrate, denatured carbonic anhydrase (data not shown). Immobilized foldases (DsbA and hPPIase) were assayed on the basis of their enzymatic activities. Immobilized DsbA was tested for its ability to catalyze protein disulfide bond formation. An RNase A with scrambled disulfide bridges was incubated with DsbA-Sepharose in the presence of excess GSH over GSSG. The recovery of RNase A activity was significantly higher in the presence of DsbA-Sepharose than the case with SP Sepharose without DsbA and the immobilized DsbA effect was concentration-dependent (Fig. 3A). Immobilized hPPIase assay was carried out based on the observation that chymotrypsin cleaved the C-terminal amide bond only in the *trans *X-Pro conformer of the chromogenic substrate, X-Pro-Phe-*p*-nitroanilide (X is any amino acid) [17]. Immobilized hPPIase can catalyze the *cis-trans *interconversion of the Ala-Pro bond in the substrate N-Suc-AAPF-*p*-nitroanilide compared to the level of spontaneous isomerization in the absence of immobilized hPPIase (Fig. 3B). The burst phase due to the cleavage of the excess *trans *isomer is completed during the mixing time and the remaining absorbance change is due to the *cis-trans *isomerization catalyzed by the immobilized hPPIase (Fig. 3B).

### Batchwise refolding of denatured and reduced proteins

The immobilized folding machineries were found to facilitate the refolding of denatured and reduced RNase A with high efficiency (Table 1). The refolding efficiency is the ratio of the soluble fraction to the total amount of denatured and reduced protein used for refolding and the functional recovery is estimated from the specific activity of the recovered soluble protein relative to that of native enzyme. The hPPIase-Sepharose used for the refolding of RNase A generated a 67% refolding yield, while 17.2% was observed using SP Sepharose Fast Flow alone. The mini-chaperone-Sepharose and DsbA-Sepharose gave refolding yields of about 55% and 58%, respectively (Table 1). The binary refolding matrices showed similar refolding yields to single refolding matrices. The binary refolding matrix of mini-chaperone/DsbA-Sepharose gave a high yield of protein folding, which was corresponding to 68% efficiency. The ternary matrix (mini-chaperone/DsbA/hPPIase-Sepharose) gave the highest yield of 73% among all kinds of the refolding matrices. The RNase A prior to the denaturation and reduction initially had 76% of the activity of the purest protein under our assay condition. Further, the maximum activity recovery was 31% more than that initially present. Thus, the refolding matrices had reconditioned the RNase A by converting inactive protein into active. Reconditioning activity has been proposed as a general property of refolding systems using immobilized foldases [13,16]. We have applied the same procedure to another protein, cyclohexanone monooxygenase (CHMO) expressed as inclusion bodies from recombinant *E. coli *BL21(DE3). More than 99% activity yield was obtained on batchwise refolding of CHMO with refolding matrices. The refolding efficiency of CHMO inclusion body using the folding machineries displayed on the cation exchanger was also shown in Table 1. The SP Sepharose Fast Flow by itself produced less than 2.1% of refolding yield, which was all aggregated. Among single refolding gels, mini-chaperone-Sepharose showed the highest refolding yield of 31%. Binary refolding matrices exhibited better results than single refolding matrices. The mini-chaperone/hPPIase-Sepharose generated the best refolding yield of 46% in the group of binary refolding matrix. The ternary matrix (mini-chaperone/DsbA/hPPIase-Sepharose) gave the highest yield of 53% among all types of the refolding matrices. Similar to RNase A, the native CHMO used for comparison of activity recovered from refolding matrices initially had 80% of the activity of the purest protein under our assay condition. Moreover, the highest recovery of CHMO activity was 25% more than that initially present, suggesting the reconditioning of CHMO activity by refolding matrices immobilized folding machineries. No leakage of folding machineries from the cation exchanger was observed during the refolding process, which was confirmed by SDS-PAGE (data not shown).

**Table 1:** Batchwise refolding of denatured and reduced RNase A and CHMO.

		**RNase A**		**CHMO**	
		
**Refolding condition**		Refolding yield* (%)	**Activity yield^† ^(%)**	**Refolding yield (%)**	**Activity yield (%)**
SP Sepharose Fast Flow alone (control)		17.2 ± 1.4	33 ± 3	2.1 ± 0.7	17 ± 2
Single refolding matrix	Mini-chaperone	55.0 ± 2.1	98 ± 5	31.0 ± 1.6	107 ± 6
	DsbA	57.6 ± 2.4	121 ± 12	8.5 ± 1.3	99 ± 3
	hPPIase	67.0 ± 3.3	115 ± 9	11.0 ± 2.2	103 ± 8
Binary refolding matrix	Mini-chaperone + DsbA	68.0 ± 1.8	107 ± 5	27.1 ± 2.4	109 ± 11
	Mini-chaperone + hPPIase	56.7 ± 2.7	118 ± 7	46.2 ± 1.8	123 ± 8
	DsbA + hPPIase	58.4 ± 2.6	126 ± 2	13.8 ± 1.5	111 ± 10
Ternary refolding matrix	Mini-chaperone + DsbA + hPPIase	73.0 ± 1.5	131 ± 3	53.0 ± 2.7	125 ± 4

Data were collected from three independent refolding experiments with identical protein concentration.

* Refolding yield: the ratio of the soluble protein to the total amount of denatured and reduced protein used for refolding.

^†^Activity yield: the specific activity of the recovered soluble protein relative to that of native RNase A or CHMO showing 76% or 80% of the specific activity of the purest protein obtained from Sigma Company

### CD analysis of refolded proteins

**Figure 4:**
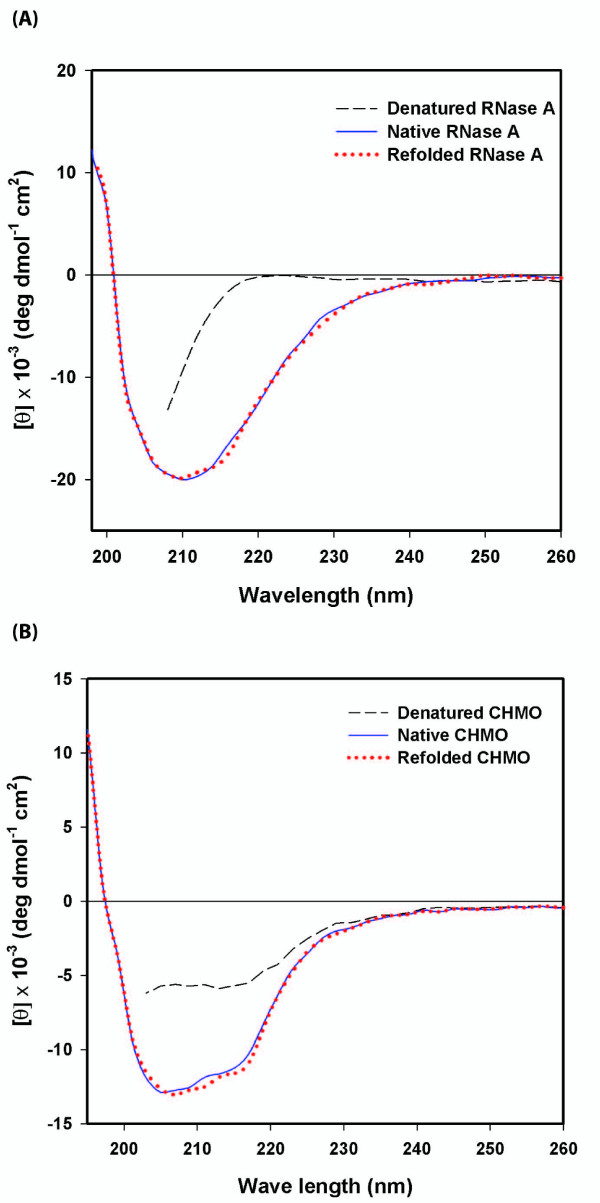
**CD spectra of RNase A and CHMO refolded with the ternary refolding matrix (mini-chaperone, DsbA and hPPIase)**. The CD spectra refolded RNase A (A) and CHMO (B) were compared with those of native and denatured and reduced RNase A and CHMO, respectively. The CD spectra were obtained using a JASCO J-715 in a quartz cuvette with 1 mm path length. RNase A and CHMO refolded by the ternary refolding gel was dissolved in 25 mM sodium phosphate buffer, pH 7.0 at 0.05 mg/ml.

The CD spectrum of refolded RNase A showing a minimum at 210 nm in the far ultraviolet (UV) region indicated that the refolded RNase A has a native-like secondary structure after comparison with the native RNase A (Fig. 4A). The denatured RNase A in 6 M GdnHCl, which has an unfolded conformation, exhibited a distinctly different CD spectrum. In the far UV-CD region, the spectrum of refolded CHMO showed two minima at 205 nm and 215 nm and it was observed to overlay very closely with that of native CHMO (Fig. 4B), suggesting that the refolded CHMO has a very comparable secondary structure to the soluble and native CHMO. This CD spectrum of the refolded CHMO is similar to that of native CHMO of another group [18].

## Discussion

Protein aggregation during production and refolding from the inclusion body of many industrial and pharmaceutical proteins in *E. coli *has been a major technical and economical bottleneck that has spurred basic research as well as process development [19]. Among various approaches to overcome protein aggregation in refolding processes, refolding chromatography systems mimicking *in vivo *folding systems are introduced as an efficient and simple way to renature proteins in high yields [13,16]. However, refolding reaction stoichiometry requires a considerable amount of folding machineries [20]. Therefore, a cost-efficient purification and immobilization of folding machineries is a critical step in order to apply this refolding methodology to a large bioprocess scale. Previously, protein disulfide isomerase (PDI) has been immobilized only in low yield and is not active [21]. It is mainly due to very reactive thiol groups in PDI. Altamirano *et al*. [16] reported the first successful immobilization of DsbA as a fully functional form. They could immobilize the DsbA on agarose by transiently blocking the active site by cyanylation. However, this technique is still challenging to repeat and cost-inefficient. In this study, we report a simple method that enables simultaneous purification and immobilization of folding machineries in fully functional forms. Among a variety of immobilization methods, adsorption is simpler and less expensive, with minimal chemical requirements and less likelihood of enzyme denaturation. However, the weak nature of the binding forces can cause leakage of the enzyme with changes in pH, ionic strength and/or temperature. In order to prevent leakage of proteins from the solid support and to exploit the simple adsorption method, the folding catalysts were charged by attaching 10-arginine amino acids. Specifically, the charged consecutive Arg_10_-stretch was genetically fused to the C-terminal of the mini-chaperone, DsbA and hPPIase to generate the charge-added folding catalysts. The generated folding catalysts were analyzed for their purification and immobilization characteristics. A single ion-exchange chromatography was performed for the one-step purification of the soluble folding machineries. Most intracellular proteins of *E. coli *have their isoelectric points (pI) at weak acidic pH in analysis of the wild type *E. coli *proteome [22]. Ninety-seven percent of intracellular proteins have pI values below pH 8.4, the same pH value of loading buffer, with only 3% at basic pH. It indicates that more than 97% *E. coli *proteins are negatively charged at pH 8.4 and cannot be bound on a cation exchanger. The calculated pI values of mini-chaperone, DsbA and hPPIase with Arg_10 _stretch was 9.43, 9.41 and 10.23, respectively, implying that the folding machineries have positive charges in 50 mM sodium phosphate buffer, pH 8.4. Therefore, they were easily purified with high purity form the negatively charged intracellular *E. coli *proteins using a single step of cation exchange chromatography. Further, Arg_10_-tail provided a simple and firm strategy for the immobilization of folding machineries. Efficient immobilization of folding machineries on a cation exchanger was achieved using an adsorption based on electrostatic interaction. The maximum amounts of the immobilized folding machinery reached 10.8 – 12.2 mg/ml and were higher than those of other groups reported previously [16]. The immobilized mini-chaperone and foldases were fully functional when assayed in a batch mode. The targeted benefit of immobilization of folding catalysts is easy separation of the refolded protein from the other components and the immobilization procedure increases the efficiency of the folding machineries, since higher concentrations of the active folding machineries can be achieved in the gel than in solution.

Recombinant proteins of industrial and pharmaceutical interest may have disulfide bonds and many are difficult to refold. After successful purification and immobilization of folding catalysts, we applied immobilized mini-chaperone and foldases to refold the cysteine- and proline-rich proteins. The first model protein is bovine pancreatic RNase A consisting of 124 amino acid residues and contains 8 cysteine residues involving in formation of 4 intramolecular disulfide bonds and 5 proline residues. The remarkable renaturation was achieved when the unfolded RNase A was treated with the individual immobilized folding machinery, suggesting that the immobilization of folding machineries on a cation exchanger through 10-arginine fusion did not inhibit their chaperone activity. This result contrasts with the spontaneous renaturation by the SP Sepharose Fast Flow in refolding buffer (Table 1). In addition, combination of the appropriate amounts of each gel makes it easier to find the optimal refolding conditions of the other target proteins because each gel has a similar refolding efficiency. The complete refolding matrices (mini-chaperone/DsbA and mini-chaperone/DsbA/hPPIase) proved to be highly efficient (68% and 73%, respectively) in restoring the native structure and biological properties of the RNase A. The synergistic effect of DsbA with PPIase and of GroEL with PDI has been observed *in vivo *and *in vitro*, indicating that they play cooperative roles in protein refolding [23,24]. The refolded RNase A recovers a native-like secondary structure based on its similar CD spectrum to that of native RNase A. The second model protein, CHMO derived from *Acinetobacter *sp. is one of the oxidative enzymes of special interest as a biocatalyst in the NADPH-dependent oxidation of cyclohexanone to ε-caprolactone *via *a Baeyer-Villiger mechanism. It comprises 543 amino acids and contains 5 cysteines and 22 prolines. When the disulfide bonds prediction was performed using *DiANNA*, which is recent state-of-the-art web-based software determining the cysteine oxidation state and disulfide connectivity of a protein [25], the result showed that 4 of 5 cysteine residues might be involved in the formation of two intramolecular disulfide bonds (Cys_330_-Cyss_475 _and Cys_376_-Cys_520_). The CHMO deposited as inclusion bodies in recombinant *E. coli *could be refolded with 53% yield using the ternary refolding matrices (mini-chaperone/DsbA/hPPIase-Sepharose) while the negligible refolding yield was achieved using the SP Sepharose resin alone. The other refolding matrices also assisted the batchwise mode of CHMO refolding (Table 1).

## Conclusion

This work demonstrates that a commonly used and relatively inexpensive ion-exchange matrix can be used for immobilization of folding catalysts. Refolding was not inhibited by protein-matrix interactions thus preventing the need for tagged protein in an affinity based method. Refolded protein was directly obtained from the refolding matrix in a highly concentrated form and free from incorrectly or incompletely refolded protein. This indicates that the proposed method can contribute significantly to bioprocess intensification as it integrates the reducing-agent removal, refolding, concentration and purification unit operations used in dilution refolding into an easily automated process. Therefore, the method is an interesting alternative for large-scale or preparative oxidative refolding of complex and highly disulfide-bonded proteins. Results reported here showed an effective refolding technique for proline- or cysteine-rich proteins obtained from *E. coli *inclusion bodies, which might be potential in the biotechnology industry.

## Methods

### Materials

Oligonucleotides were synthesized by Bioneer (Daejeon, Korea). All restriction enzymes were from New England Biolabs. The vector pET-29b(+) was obtained from Novagen.*E. coli *DH5α and BL21(DE3) cells were used for maintenance of plasmids and for the production of recombinant proteins, respectively. Plasmid, gel extraction and PCR purification kits were purchased from Qiagen. RNase A type XII-A from bovine pancreas, RNase A with scrambled disulfide bonds, cyclohexanone monooxygenase (CHMO) of *Acinetobacter *sp. expressed in recombinant *E. coli*, cytidine 2', 3'-cyclic monophosphate (2', 3'-cCMP), N-Succinyl-Ala-Ala-Pro-Phe-*p*-nitroanilide (N-Suc-AAPF-*p*-nitroanilide) and α-chymotrypsin (α-CT) were purchased from Sigma-Aldrich. All other chemicals unless otherwise stated were of reagent grade.

### Construction of template plasmid with Arg_10_-tag

Standard cloning procedures were performed according to Sambrook and Russell [26]. In order to construct the template plasmid with an Arg_10_-tail, mixture of complementary oligonucleotides (R10A and R10B) were heated to 90°C and cooled slowly to room temperature. The annealed double-stranded DNA was subjected to kination as follows: total 30 μl of reaction solution containing 20 μl of 100 pmoles/μl DNA, 3 μl of 10 × polynucleotide kinase buffer, 3 μl of 10 mM ATP, 1 μl of T4 polynucleotide kinase and 3 μl of distilled deionized water was incubated at 37°C for 1 hr and heat-inactivated at 75°C for 20 min. Phosphorylated DNA was inserted to the pET-29b(+) fragment which had been digested by BamHI and XhoI followed by dephosphorylation with calf intestinal phosphatase at 37°C for 1 hr. The resulting template plasmid that contains the 10-arginine residues was named by pETR10. The oligonucleotide primers used in this study are shown in Table 2.

**Table 2:** Oligonucleotide sequences used in this study.

**Name**	**Sequence of oligonucleotide**
R10A	5'-GATCCCGTCGCCGTCGCCGTCGCCGTCGCCGTCGCTACTAAC-3'
R10B	5'-TCGAGTTAGTAGCGACGGCGACGGCGACGGCGACGGCGACGG-3'
DsbA5	5'-TATCAACATATGAAAAAGATTTGGCTGGC-3'
DsbA3	5'-GCATTGGGATCCTTTTTTCTCGGACAGATATTTC-3'
HPPI5	5'-TATCAACATATGGTCAACCCCACCGTG-3'
HPPI3	5'-GCATTGGGATCCTTCGAGTTGTCCACAGTCAG-3'
Mini5	5'-TATCAACATATGGAAGGTATGCAGTTCGACC-3'
Mini3	5'-GCATTGGGATCCACGGCCCTGGATTGCAGC-3'

### Construction of expression plasmids

To construct expression vectors for folding machineries with C-terminal Arg_10_-tag, genes for mini-chaperone, DsbA and hPPIase were amplified by PCR from the chromosomal DNA of *E. coli *K-12 or from the cDNA of hPPIase gene as template. The primer sets used for amplification of folding machineries are as follows: Mini5 and Mini3 for mini-chaperone; DsbA5 and DsbA3 for DsbA; HPPI5 and HPPI3 for hPPIase (Table 2). The amplified DNA fragments were digested by NdeI and BamHI and cloned into the template vector pETR10 digested by the same restriction enzymes. The resulting 3 expression vectors are named as pMiniELR10, pDsbAR10, and pHPPIR10, respectively.

### Expression and purification of folding machineries

In order to prepare the folding machineries, *E. coli *BL21(DE3) cells harboring expression vectors for folding machineries were cultured in 200 ml Luria-Bertani (LB) medium (0.5% yeast extract, 1% tryptone and 1% NaCl) supplemented with 50 μg/ml kanamycin at 30°C and induced with 0.5 mM isopropyl-β-thiogalactopyranoside (IPTG) at an OD_600 _of 0.5. After 6 hr induction, cells were harvested by centrifugation at 8000 × g for 30 min at 4°C. The harvested cells were resuspended in 20 ml of 50 mM sodium phosphate buffer, pH 8.4 and then disrupted by French Press (Thermo Spectronic) at 20,000 psi in an ice bath. After removing cell debris by centrifugation, the resulting supernatant was directly loaded onto an ion-exchange column for purification. An automated chromatography system, ÄKTA prime (GE Healthcare Life Sciences) was used to monitor the protein elution peak. The ion-exchange column, HiTrap Sepharose Fast Flow (GE Healthcare Life Sciences) loaded with *E. coli *cell extracts was washed with 10 column volumes of 50 mM sodium phosphate buffer, pH 8.4 at a flow rate of 1.0 ml/min. The unbound proteins were washed out at this step. Continuous linear gradient from 0 to 1 M NaCl was applied to separate the proteins bound to the cation exchanger. The absorbance at 280 nm and the conductivity in the eluent were observed. The final eluted fractions were collected and subjected to further analyses.

### Protein quantification and detection

Protein concentration was determined by the Bradford method using the protein assay kit (Bio-Rad) according to the manufacturer's instructions. Protein samples were analyzed by SDS-PAGE using 12% gels and detected by staining the gels with Coomassie brilliant blue R-250. The dried SDS-PAGE gels were imaged and digitized with a high resolution scanner (Scanjet ADF, Hewlett Packard). The quantification of bands intensity and purity was carried out using the densitometry software (TotalLab 1.01, Nonlinear Dynamics Ltd.).

### MALDI-TOF analysis of purified folding machineries

Mass spectra of purified folding catalyst proteins were collected by MALDI-TOF (Voyager-DE Biospectrometry Workstation). The purified folding machinery proteins were mixed with 3, 5-dimethyl-4-hydroxycinnamic acid as a matrix, dropped on a sample plate and air dried until crystallization occurred. Then the sample plate was loaded and analyzed the molecular weight at the proper conditions.

### Preparation of denatured and reduced model proteins

For the preparation of denatured and reduced CHMO,*E. coli *BL21(DE3) cells containing pMM4 for CHMO expression [27] were grown at 37°C in LB supplemented with 100 μg/ml ampicillin. Expression was induced by adding IPTG to a final concentration of 1 mM when the culture was grown to OD_600 _of about 0.5. After induction for 6 hr, cells were harvested by centrifugation at 8000 × g for 30 min and resuspended in lysis buffer (10 mM Tris-HCl, 1 mM EDTA and 100 mM monobasic sodium phosphate, pH 8.0). After the cells were disrupted by being passed two times through the French Press at 20,000 psi, cell lysates were centrifuged at 12000 × g for 30 min to remove soluble proteins of *E. coli *and the precipitates were resuspended in washing buffer (2% Triton X-100, 20 mM EDTA and 100 mM monobasic sodium phosphate, pH 8.0). This step was repeated three times to wash out residual soluble proteins. The washed insoluble pellet was defined as CHMO inclusion body. The CHMO inclusion body and RNase A type XII-A (Sigma-Aldrich) were denatured and reduced in a solution containing 6 M guanidine hydrochloride (GdnHCl), 0.14 M DTT and 2 mM EDTA in 50 mM MOPS buffer, pH 8.0. Each solution was incubated for at least 12 hr at 25°C. The denaturation buffer was prepared just prior to use. The activities of denatured and reduced CHMO and RNase A were measured to confirm their fully denatured and reduced states.

### Enzymatic activity assay

For the enzyme activity assay of RNase A, cleavage of phosphodiester bonds was monitored. RNase A (5 – 100 μl of a stock solution in 100 mM Tris-acetate buffer, pH 8.0) was added to solutions containing 2', 3'-cCMP, GdnHCl, EDTA and DTT in 50 mM Tris-acetate buffer, pH 8.0 to give a final volume of 400 μl with 4.5 mM 2', 3'- cCMP, 0.6 M GdnHCl, 0.2 mM EDTA and 14 mM DTT. The changes in absorbance were followed in thermostatic quartz cuvette (1 cm length) at 296 nm using an Ultrospec 4000 spectrophotometer (GE Healthcare Life Sciences). CHMO activity was determined from the rate of NADPH consumption upon the addition of cyclohexanone to the reaction mixture [28]. The assay mixture contains, in a final volume of 400 μl, 40 μl of 4.0 mM NADPH, 20 μl of crude enzyme solution with 0.01 – 0.2 units of activity and 330 μl of 0.1 M Glycine/NaOH buffer, pH 9.0. An endogenous rate of NADPH consumption was measured by pre-incubation at 30°C in the thermostatic cell holder of the spectrophotometer for 2 min prior to the addition of 25 mM cyclohexanone to the assay mixture. After pre-incubation, 10 μl of cyclohexanone is added and the decrease in absorbance at 340 nm is monitored. CHMO activity was calculated as the difference between the endogenous rate of cofactor consumption and the stimulated rate of NADPH oxidation. Disulfide isomerization activity of immobilized DsbA was measured as described by the previous report with some modifications [29]. Inactive RNase A with scrambled disulfide bonds was used for its activity recovery catalyzed by the disulfide shuffling activity of DsbA. Briefly, RNase A with scrambled disulfide bonds (final concentration, 0.2 mg/ml) was incubated in 3.6 mM GSH, 0.9 mM GSSG, 1 mM EDTA and 100 mM Tris-acetate buffer pH 8.0 in the presence or absence of immobilized DsbA. This reaction solution was mixed with 360 μl of 2', 3'-cCMP (0.1 mg/ml) in 0.1 M MOPS buffer, pH 7.0 and incubated at 25°C for 5 min. After gentle mixing, the cuvette was placed in the spectrophotometer for 20 min to allow thermal equilibration of the solution to 25°C. Hydrolysis of 2', 3'-cCMP was measured by the increase in absorption at 296 nm. The hPPIase activity was determined by a coupled assay with α-CT using the short synthetic peptide substrate, N-Suc-AAPF-*p*-nitroanilide in 88% *trans*, 12% *cis *configuration [17]. The hPPIase activity was measured as the *cis-trans *isomerization of the alanine-proline peptide bond in the peptide. The assay relies on the inability of α-CT to hydrolyze N-Suc-AA-*cis*-PF-*p*-nitroanilide. The substrate will be rapidly hydrolyzed to N-Suc-AA-*trans*-PF-*p*-nitroanilide and released *p*-nitroanilide is quantified spectrophotometrically at 390 nm. The assay buffer (940 μl of 20 mM sodium phosphate buffer pH 8.0), 50 μl of gels displayed with the hPPIase protein (1.2 nmol) and the peptide substrate (20 μl of 4 mM stock solution dissolved in DMSO; final concentration 70 μM) were pre-equilibrated in the spectrophotometer for 10 min. Immediately before the assay was started, α-CT (30 μl of 2.24 mM stock solution in 1.0 mM HCl; final concentration 75 μM) solution was added. The final volume in a masked, semi-micro 1-cm path length cell was 1.0 ml. After a delay from the onset of mixing, the absorbance of *p*-nitroanilide was followed at 390 nm until the reaction was complete. Absorbance readings were collected on the spectrophotometer interfaced to a computer, by use of commercial data acquisition software (SWIFT, GE Healthcare Life Sciences).

### Construction of refolding matrices

A cation exchanger (SP Sepharose Fast Flow) was pre-equilibrated with 50 mM sodium phosphate buffer, pH 8.4. Each folding catalyst (mini-chaperone, DsbA and hPPIase) in the same buffer was separately added to the pre-equilibrated cation exchanger and mixed for at least 2 hr with gentle shaking. The resulting refolding gels immobilized with each protein (mini-chaperone-Sepharose, DsbA-Sepharose and hPPIase-Sepharose) were thoroughly mixed in equal molar ratios of the proteins to make binary and ternary refolding gels. The unbound proteins were subsequently removed by washing with 50 mM sodium phosphate buffer, pH 8.4. In order to determine the binding capacity of folding machineries into the cation exchanger, each refolding gel immobilized with folding machineries was mixed with 2 bead volumes of 50 mM sodium phosphate buffer, pH 8.4 containing 1 M NaCl. After gentle mixing for 30 min at 25°C, the solid bead was eliminated by centrifugation. The supernatant was then subjected to the determination of protein. This procedure was repeated twice, which yield virtually the same protein concentration bound to SP Sepharose Fast Flow within the experimental error range of ± 5%.

### Refolding of RNase A and CHMO

The refolding of denatured and reduced CHMO and RNase A was performed according to the method of Altamirano *et al*. [16]. Refolding was initiated by equilibration of the refolding gels with refolding buffer containing 50 mM MOPS buffer, pH 8.0, 1.0 mM GSH, 0.2 mM GSSG and 14 mM DTT. In all cases, the denatured and reduced model proteins (100 μg in 40 μl) were very slowly mixed and diluted 100-fold with a resuspension of the refolding matrix and kept at 25°C under gentle mixing. After incubation for 10 min, the gel suspension was then centrifuged at 6000 × g for 10 min to separate the supernatant. The supernatants (soluble proteins) were eventually concentrated and desalted by Vivaspin concentrators (vivaspin 500; 5,000 MWCO PES, VivaScience) according to the manufacturer's instruction. The concentration and enzyme activity of refolded protein in the supernatant were determined and the amount of soluble protein was subtracted from the total proteins subjected to the initial refolding procedure to estimate the amount of aggregation. Control experiments were performed in which the denatured and reduced model proteins were added to the SP Sepharose Fast Flow equilibrated with refolding buffer alone.

### Circular dichroism (CD) spectroscopy

To examine the correct folding of RNase A and CHMO, the RNase A and CHMO refolded by the ternary refolding gel were dissolved in 25 mM sodium phosphate buffer, pH 7.0 at 0.05 mg/ml. The far UV CD spectra of refolded RNase A and CHMO were monitored between 3 and 6 min at from 190 to 260 nm with a JASCO J-715 spectro-polarimeter (JASCO) using a quartz cuvette with 1 mm path length. CD spectra of native and denatured and reduced RNase A and CHMO were also recorded. Spectra were corrected by subtracting the buffer baseline and were averaged over 10 scans for far UV CD measurements.

## Authors' contributions

DHL drafted the manuscript and participated in the design and coordination of the study. DHK helped to draft the manuscript and participated in the design of both the molecular variants and the study. SGK performed the immobilization experiments. JHS conceived the molecular design, organized funding and helped to draft the manuscript, designed and coordinated the study. All authors read and approved the final manuscript.
